# Physical Activity Levels Among Older Adults in Urban Central Asia: A Cross-Sectional Study

**DOI:** 10.3390/healthcare14131843

**Published:** 2026-06-24

**Authors:** Yerkezhan Tolegenova, Aigul Abduldayeva, Ainur Aiypkhanova, Gulnur Doszhanova, Olzhas Kozhamkulov

**Affiliations:** 1 Scientific Research Institute of Preventive Medicine Named After Y.D. Dalenov, Astana Medical University, Astana 010000, Kazakhstan; tolegenova.y@amu.kz (Y.T.); doszhanova.g@amu.kz (G.D.); kozhamkulov.o@amu.kz (O.K.); 2 Indiana Department of Health, Indianapolis, IN 46204, USA; aiaiypkh@gmail.com

**Keywords:** physical activity (PA), older adults, healthy aging, PASE, body composition, visceral fat, muscle strength, urban health, Central Asia, cross-sectional study, health promotion, physical activity interventions

## Abstract

Background: Physical activity is a key modifiable factor influencing healthy aging, yet data on activity patterns and their physiological correlates in older adults from Central Asia remain limited. Understanding these relationships is essential for informing region-specific health promotion strategies. Objectives: This study assessed physical activity levels among urban-dwelling older adults in Astana, Kazakhstan, and examined associations between activity level, body composition, visceral fat accumulation, metabolic indicators, and muscle strength. Methods: A cross-sectional study was conducted among 608 adults aged ≥60 years (median age: 68 years; 82.1% women). Physical activity was measured using the validated Physical Activity Scale for the Elderly (PASE). Anthropometric and body composition indicators, including BMI, total and visceral fat, skeletal muscle mass, and handgrip strength, were evaluated. Spearman correlation and linear regression analyses were applied. The analyses were exploratory and did not include adjustment for potential confounders such as sex, chronic disease burden, or socioeconomic status; therefore, the observed associations should be interpreted with caution. Results: The median PASE score was 55.55, with 61.8% of participants demonstrating moderate activity levels, primarily through walking and household tasks. In analyses without adjustment for potential confounding factors, PASE scores showed weak inverse associations with visceral fat (ρ = −0.214; *p* < 0.001) and waist-to-hip ratio (ρ = −0.154; *p* < 0.001), as well as weak positive associations with handgrip strength. Across the reported significant associations, correlation coefficients ranged from |ρ| = 0.103 to 0.235, and the explanatory capacity of the regression models was low, with R^2^ values ranging from 0.6% to 8.2%. Conclusions: Higher habitual physical activity may be linked to selected bioelectrical impedance parameters, WHR, and handgrip strength among urban older adults. Given the cross-sectional design, causal interpretation should be approached with caution. These findings provide meaningful regional baseline evidence for future longitudinal and intervention studies on physical activity and healthy aging in Central Asia.

## 1. Introduction

Physical activity (PA) is a key modifiable determinant of healthy aging and is associated with lower mortality and reduced risk of major noncommunicable disease outcomes in older adults [1,2,3,4,5]. Recent step-count and cohort evidence indicates that higher daily movement is associated with lower all-cause mortality, with benefits observed even below the commonly cited threshold of 10,000 steps per day [1,2,3,4]. Prospective evidence also suggests that daily step counts and step intensity are associated with cardiovascular disease, cancer outcomes, and all-cause mortality [5]. These findings support the importance of regular, feasible daily activity for older adults, including walking and other low-barrier forms of movement.

Beyond epidemiological outcomes, recent studies also suggest that PA may be linked to biological aging processes. Physical activity has been associated with slower epigenetic aging [6], while DNA methylation-based biomarkers such as GrimAge and DunedinPACE have been increasingly used to characterize aging trajectories and age-related health risks [7,8]. Recent evidence also indicates that exercise may have favorable effects on telomere length and skeletal muscle mitochondrial function, although the magnitude of these effects may vary by exercise modality, duration, and population characteristics [9,10].

Global policy guidance reflects this evidence. The WHO 2020 Guidelines on Physical Activity and Sedentary Behaviour recommend that adults, including older adults, engage in regular aerobic physical activity and reduce sedentary behavior. For older adults, multicomponent activities that include balance and strength training are particularly important for maintaining functional capacity and preventing falls [11].

Despite robust international evidence, context-specific data from Central Asia appear to remain limited, particularly for urban-dwelling older adults. In this region, walkability, climate, transport infrastructure, social services, and cultural roles may influence physical activity patterns and feasibility. Region-specific evidence is therefore needed to clarify patterns of daily activity and their relationship with body composition and functional indicators in older adults from Central Asia.

Body composition profiling and functional assessment are important for evaluating health risks in aging populations. Indicators such as body mass index, waist-to-hip ratio, visceral fat, skeletal muscle mass, and handgrip strength help characterize adiposity, metabolic vulnerability, and functional capacity. Bioelectrical impedance analysis is widely used in community-based studies and offers a practical approach to assessing body composition in older adults. However, its interpretation should account for device-specific algorithms as well as age- and sex-related differences [12].

For PA measurement, the Physical Activity Scale for the Elderly (PASE) is a widely used instrument designed to capture leisure-time, household, and occupational activities in older adults. Recent evidence supports the use of PASE in community-dwelling older adults, while also emphasizing the need for caution when interpreting self-reported physical activity.

Available PASE-based evidence remains sparse outside North America and high-income countries. A recent scoping review identified 232 PASE studies from 35 countries; however, nearly half were conducted in North America and most were conducted in high-income settings [13].

This study assessed physical activity levels among urban-dwelling older adults in Astana, Kazakhstan, and examined associations between PA, body composition, visceral fat accumulation, metabolic indicators, and muscle strength. Our main hypothesis was that higher physical activity levels, assessed using PASE, would be associated with more favorable body composition and functional indicators among older adults. Specifically, we hypothesized that higher PASE scores would be associated with lower BMI, lower total body fat percentage, lower visceral fat level, lower waist-to-hip ratio, and greater handgrip strength. Although the cross-sectional design does not allow causal inference, this study provides regional baseline data to support future longitudinal and interventional research and to inform health promotion strategies in Central Asia.

### Study Objective

This study assessed physical activity levels among urban-dwelling older adults in Astana, Kazakhstan, and examined associations between activity level, body composition, visceral fat accumulation, metabolic indicators, and muscle strength. The primary outcome of this study was the total physical activity level assessed using the Physical Activity Scale for the Elderly (PASE). Secondary outcomes included BMI, total body fat percentage, visceral fat level, waist-to-hip ratio, skeletal muscle mass, metabolic age, and handgrip strength. The analyses were exploratory in nature and aimed to identify associations between physical activity and selected anthropometric, body composition, and functional indicators.

## 2. Materials and Methods

### 2.1. Participants

A total of 608 community-dwelling and institutionalized adults aged 60–89 years were included. Participants were recruited from two settings: (1) Social Service Centers, which provide long-term residential care for older individuals with reduced functional autonomy; and (2) Active Longevity Centers, attended voluntarily by older adults living independently in their own homes. Individuals from the latter group generally demonstrated higher mobility and greater engagement in social and physical activities.

In total, 693 older adults were approached for participation; 608 agreed to participate and were included in the final analysis, corresponding to a response rate of 87.7%. Eighty-five individuals declined participation or were not included, resulting in a non-participation rate of 12.3%. All included participants had complete data for the variables used in the main analyses.

Eligible participants were aged 60 years or older, able to provide written informed consent, and able to complete the questionnaire and participate in anthropometric, body composition, and functional assessments. Exclusion criteria included severe cognitive impairment or inability to understand the questionnaire, severe functional limitations preventing participation in the assessments, contraindications to bioelectrical impedance analysis, and refusal to participate. No formal cognitive screening instrument was used; cognitive eligibility was assessed based on the participant’s ability to understand the study information, provide informed consent, and complete the questionnaire with or without assistance from trained research staff.

No formal sample size or power calculation was performed prior to recruitment. The study was designed as an exploratory cross-sectional investigation aimed at obtaining regional baseline data on physical activity and related body composition and functional indicators among older adults.

### 2.2. Study Design

The study used a cross-sectional design.

### 2.3. Instruments

Physical activity was assessed using the Physical Activity Scale for the Elderly (PASE), a validated self-report instrument specifically developed for older adults. The PASE captures leisure-time, household, and occupational activities performed during the previous seven days, and its total score reflects overall physical activity level. The instrument has demonstrated acceptable validity and reliability in older adult populations, including a reported test–retest reliability coefficient of 0.75 in the original validation study [14,15]. In the present study, PASE scoring followed established procedures, with higher total scores indicating higher levels of physical activity.

For descriptive interpretation, PASE scores were categorized using the preliminary age- and sex-specific normative values reported in the original PASE manual. For each sex and age group, the mean ± standard deviation range was considered the reference range and was classified as moderate physical activity. Scores below the lower limit of this range were classified as low physical activity, whereas scores above the upper limit were classified as high physical activity. Thus, the low, moderate, and high categories used in this study were operational descriptive categories based on PASE normative reference values.

Body composition was measured using a Tanita BC-545N bioelectrical impedance analyzer (Tanita Corporation, Tokyo, Japan) [16,17]. The device provided estimates of body mass index (BMI), total body fat percentage, visceral fat level in device-specific units, skeletal muscle mass, bone mass, and basal metabolic rate. Bioelectrical impedance analysis has been validated as a practical, non-invasive method for body composition assessment in older adults and is widely used in field and community-based studies. However, visceral fat values obtained using BIA were interpreted as device-derived estimates rather than direct imaging-based measurements, since computed tomography and magnetic resonance imaging remain reference methods for direct assessment of visceral adipose tissue.

Anthropometric measurements included standing height, body weight, waist circumference, and hip circumference. Waist-to-hip ratio (WHR) was calculated as waist circumference divided by hip circumference.

Sociodemographic information (age, sex, employment status, chronic disease history, and living arrangement) was collected through structured interviews.

### 2.4. Procedure

Body composition measurements:

Body composition measurements were performed using a Tanita bioelectrical impedance analyzer. Participants were measured in light clothing and without shoes. Before measurement, participants were asked to remove metal objects and stand barefoot on the device according to the manufacturer’s instructions. Measurements were conducted in the morning or early afternoon to minimize diurnal variability. Participants were instructed to avoid heavy meals and excessive fluid intake shortly before the assessment; however, strict fasting status and hydration control were not applied.

Handgrip strength protocol:

Handgrip strength was assessed using a calibrated hand dynamometer (Tulinovsky Instrument-Making Plant “TVES”, Tulinovka, Tambov Region, Russia). Participants were tested in a seated position, with the shoulder adducted, the elbow flexed at approximately 90 degrees, and the forearm in a neutral position. Measurements were performed for both hands, with two trials per hand and a short rest interval between attempts. The highest value recorded across all trials was used for analysis.

Reliability statement:

All measurements were performed by trained research staff following standardized procedures. However, formal inter-rater and intra-rater reliability assessments were not conducted.

### 2.5. Statistical Analysis

Data were analyzed using IBM SPSS Statistics version 29.0. The normality of continuous variables was assessed using the Kolmogorov–Smirnov test, together with visual inspection of histograms and Q–Q plots. Normally distributed continuous variables were presented as mean ± standard deviation (M ± SD), whereas non-normally distributed variables were presented as median and interquartile range (Q1–Q3). Categorical variables were presented as frequencies and percentages.

Associations between physical activity level, assessed by the PASE score, and anthropometric, body composition, and functional indicators were evaluated using Spearman’s rank correlation coefficient (ρ). Scatter plots and Spearman correlation coefficients represented crude, unadjusted associations between PASE score and the studied variables. Simple linear regression models were constructed to further characterize the observed associations. Regression coefficients (β), 95% confidence intervals (CI), *p*-values, and coefficients of determination (R^2^) were reported. A two-sided *p*-value < 0.05 was considered statistically significant.

Missing data were handled using available-case analysis. Participants with missing data for a given variable were excluded only from analyses involving that variable. No imputation of missing values was performed.

## 3. Results

### 3.1. Sample Characteristics

A total of 608 older adults were included in the study. Most participants were recruited from Active Longevity Centers (*n* = 546; 89.8%; 95% CI: 87.1–92.1). These are daytime community-based centers attended by older adults living independently in their own homes. A smaller subgroup was recruited from Social Service Centers (*n* = 62; 10.2%; 95% CI: 7.9–12.9), which provide long-term residential care for older adults with partially reduced functional autonomy.

Women comprised 82.1% of the sample (*n* = 499; 95% CI: 78.8–85.0), and men comprised 17.9% (*n* = 109; 95% CI: 15.0–21.2). The median age was 68.0 years (Q1–Q3: 65.0–73.0). Younger-old adults (60–74 years) accounted for 81.1% of the sample (*n* = 492), whereas older-old adults (≥75 years) accounted for 18.9% (*n* = 116; 95% CI: 15.9–22.3). Descriptive clinical measures were as follows: median systolic blood pressure was 140.0 mmHg (Q1–Q3: 125.0–152.0), median diastolic blood pressure was 85.0 mmHg (Q1–Q3: 78.75–93.0), and median heart rate was 73 beats/min (Q1–Q3: 66–81). Detailed participant characteristics are presented in Table 1.

### 3.2. Anthropometrics and Body Composition

Mean BMI was 27.73 ± 4.80 kg/m^2^, with pre-obesity most prevalent (~40%) and obesity class I–III observed in ~31% overall; underweight was uncommon. Median total body fat was 37.8% (Q1–Q3: 32.6–42.6). By category, 33.9% were in the normal range, 27.0% showed excess fat, and 37.5% fell in the obesity range; fat deficit was rare (1.6%). Visceral fat (device units) had a median of 11.0 (Q1–Q3: 9.0–13.0), with 70.4% in the normal category and 29.6% elevated, indicating that central adiposity was common but not universal.

Additional parameters reflected typical aging patterns: skeletal muscle mass median 40.20 kg; total body water 44.70%; handgrip strength 19.0 kg; basal metabolic rate 1286.5 kcal/day; and metabolic age 66 years. Where assessed, A Body Shape Index (ABSI) values were stable (median ≈ 0.08), consistent with the central adiposity distribution noted above. Comprehensive distributions are provided in Table 1.

### 3.3. Physical Activity Patterns (PASE Items)

Activity structure varied substantially by type and intensity. Walking was the most common and regular activity: 76.6% walked daily, with a median duration of 60 min (Q1–Q3: 40–90), reflecting high engagement with accessible, low-risk activity.

Light recreational activities (e.g., simple exercises, slow-paced gymnastics, low-intensity games) were performed daily by 15.1% and 2–3 times/week by ~15–20% (median 45 min), indicating organized low-intensity participation among a meaningful subset.

Moderate-intensity activities (e.g., dancing, skating, more vigorous movement) were less frequent—absent in >77% overall; ~17% participated 2–3 times/week, and <2% daily—with short durations consistent with irregular practice. Vigorous activities were uncommon: 83.4% of participants reported no vigorous activity, 4–7% reported such activity two to three times per week, and fewer than 1% reported daily vigorous activity.

Strength/endurance training was infrequent: nearly 90% reported none, 5.5% performed strength work daily, and 3.1% several times per week; durations were generally brief.

**Figure 1:**
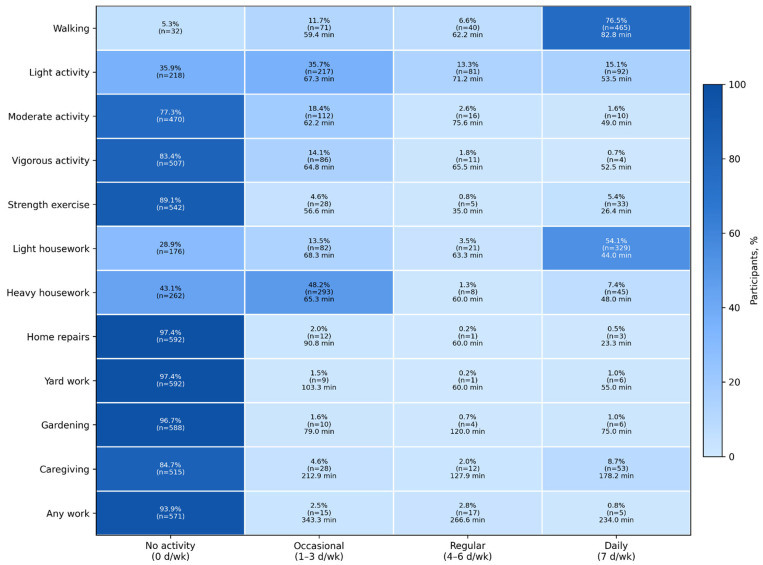
Frequencies and durations activity among older adults in Astana. Note: Each cell shows percentage and number of participants. For active groups, mean session duration is shown in minutes.

Household activities were a major contributor to daily PA: light household work was performed daily by 54.2% (absent in 28.8%), while heavy household work was absent in 43.1%, once/week in 26%, and daily in 7.4%, reflecting functional variability across the cohort. More demanding domestic tasks were rare (home repairs 97.5% absent; yard maintenance 97.4% absent; gardening 96.7% absent), and where present, episodic. Caregiving responsibilities were regular in 8.7% (absent in 84.7%). Paid/voluntary work was uncommon: 95.2% reported no work; ~1–2% worked 2–7 days/week. Frequencies and durations are summarized in Figure 1.

### 3.4. Overall Physical Activity Level (PASE Total Score)

**Figure 2:**
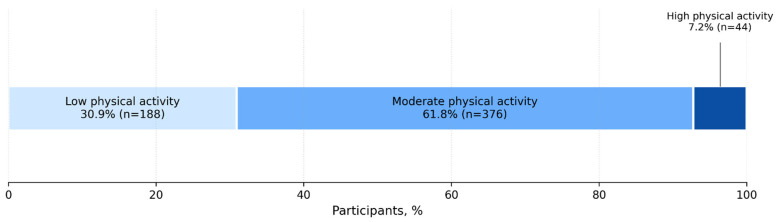
Level of physical activity according to the PASE scale. Labels show percentage and number of participants in each category.

The median PASE score was 55.55 (Q1–Q3: 34.66–89.30). Distribution: low 30.9% (*n* = 188; 95% CI: 27.3–34.8), moderate 61.8% (*n* = 376; 95% CI: 57.8–65.7), and high 7.2% (*n* = 44; 95% CI: 5.3–9.6). See Figure 2.

### 3.5. Correlation and Regression Analyses (Overall Sample)

**Figure 3:**
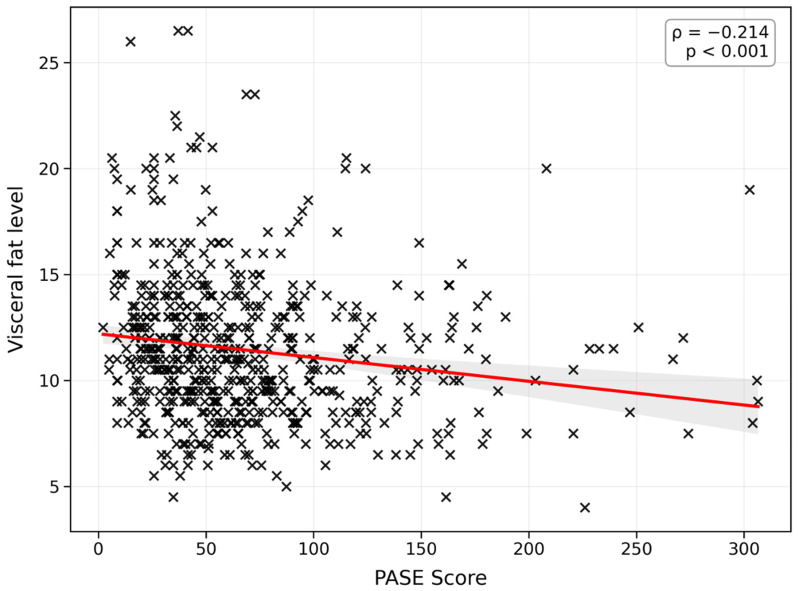
Relationship between physical activity level and visceral fat level in the overall study sample. Note: Each symbol represents an individual observation. The red solid line represents the fitted linear regression line, and the grey shaded area indicates the 95% confidence interval for the mean predicted outcome.

**Figure 4:**
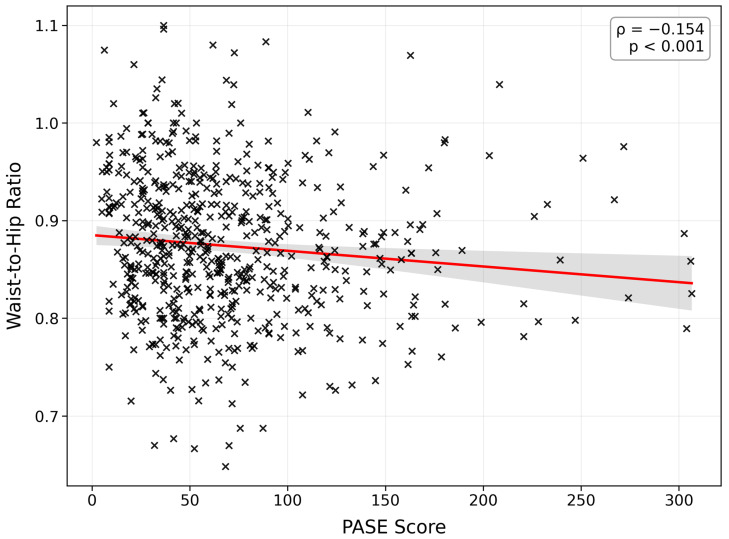
Relationship between physical activity level and waist-to-hip ratio in the overall study sample. Note: Each symbol represents an individual observation. The red solid line represents the fitted linear regression line, and the grey shaded area indicates the 95% confidence interval for the mean predicted outcome.

Higher physical activity, assessed by the PASE score, was inversely associated with visceral fat level (ρ = −0.214; *p* < 0.001) and WHR (ρ = −0.154; *p* < 0.001), indicating weak inverse associations. Models were:

Visceral fat = −0.011 × PASE + 12.198, R^2^ ≈ 2.8% (β = −0.011; 95% CI: −0.017 to −0.006) (see Figure 3);

WHR = −0.00018 × PASE + 0.885, R^2^ ≈ 1.2% (β = −0.00018; 95% CI: −0.00028 to −0.00005) (see Figure 4).

### 3.6. Sex-Stratified Results

Men (*n* = 109)

Men were older (mean 71.15 ± 5.72 years) and exhibited higher WHR (mean 0.93 ± 0.07), greater visceral fat (mean 15.26 ± 4.09), and lower PASE (median 34.83; Q1–Q3: 24.60–59.05) compared with women (see Table 2).

**Figure 5:**
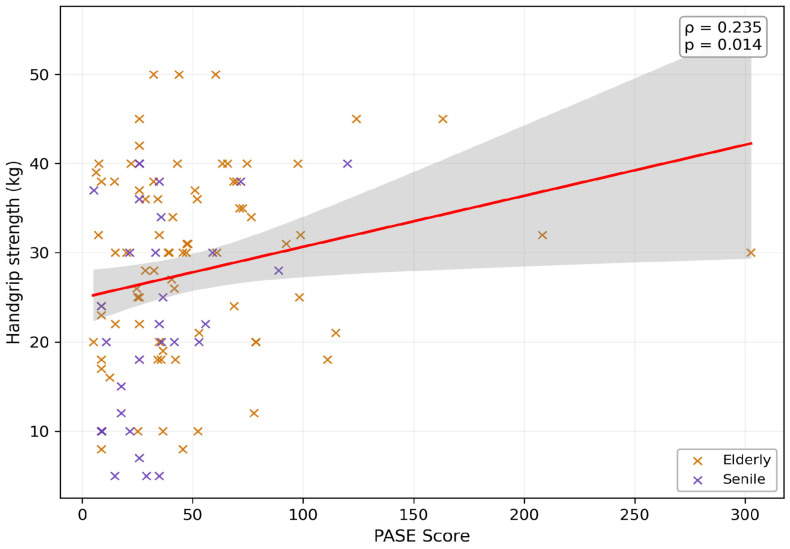
Relationship between physical activity level and handgrip strength in men. Note: Each symbol represents an individual observation. The red solid line represents the fitted linear regression line, and the grey shaded area indicates the 95% confidence interval for the mean predicted outcome.

Among men, PASE was positively associated with handgrip strength (ρ = 0.235; *p* = 0.014), indicating a weak positive association:

Handgrip strength = 0.057 × PASE + 24.94, R^2^ ≈ 4.7% (β = 0.057; 95% CI: 0.008 to 0.107) (see Figure 5).

Women (*n* = 499)

Women were younger (median 68 years; Q1–Q3: 64.0–73.0) and had higher PASE (median 61.45; Q1–Q3: 37.92–92.65). BMI and body fat distributions are shown in Table 3 (women).

**Figure 6:**
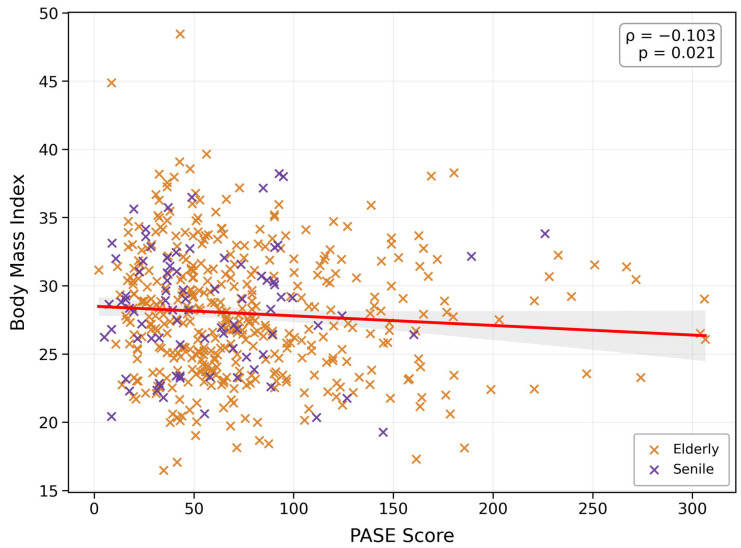
Relationship between physical activity level and body mass index in women. Note: Each symbol represents an individual observation. The red solid line represents the fitted linear regression line, and the grey shaded area indicates the 95% confidence interval for the mean predicted outcome.

**Figure 7:**
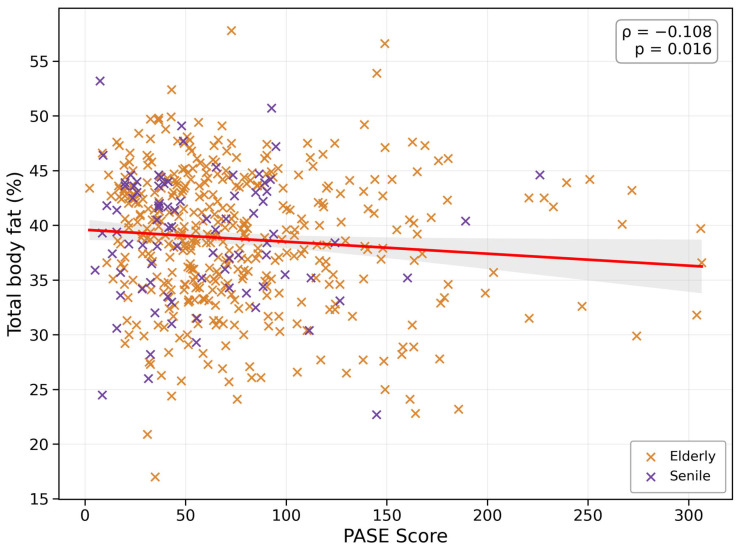
Relationship between physical activity level and total body fat in women. Note: Each symbol represents an individual observation. The red solid line represents the fitted linear regression line, and the grey shaded area indicates the 95% confidence interval for the mean predicted outcome.

**Figure 8:**
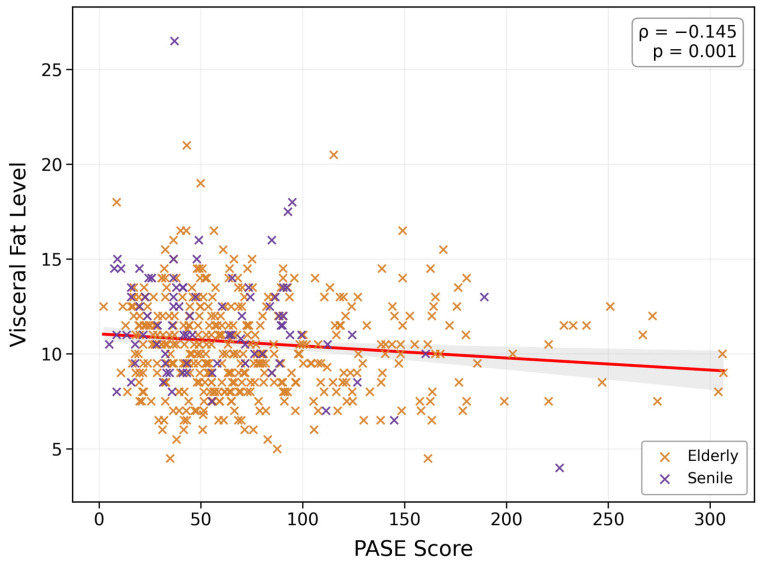
Relationship between physical activity level and visceral fat level in women. Note: Each symbol represents an individual observation. The red solid line represents the fitted linear regression line, and the grey shaded area indicates the 95% confidence interval for the mean predicted outcome.

Among women, PASE correlated inversely with BMI (ρ = −0.103; *p* = 0.021), total body fat (ρ = −0.108; *p* = 0.016), and visceral fat (ρ = −0.145; *p* = 0.001), indicating weak inverse associations. Models were:

BMI = −0.007 × PASE + 28.50, R^2^ ≈ 0.63% (β = −0.007; 95% CI: −0.015 to 0.001) (see Figure 6);

Total body fat (%) = −0.011 × PASE + 39.591, R^2^ ≈ 0.9% (β = −0.011; 95% CI: −0.021 to −0.001) (see Figure 7);

Visceral fat = −0.006 × PASE + 11.054, R^2^ ≈ 1.5% (β = −0.006; 95% CI: −0.011 to −0.002) (see Figure 8).

**Figure 9:**
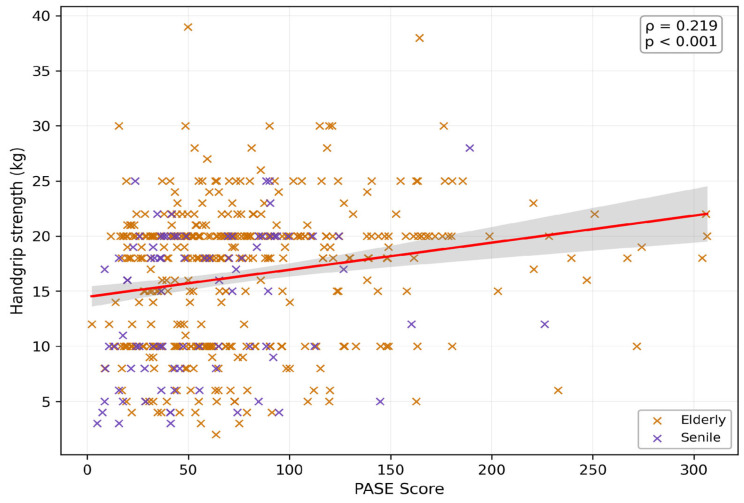
Relationship between physical activity level and handgrip strength in women. Note: Each symbol represents an individual observation. The red solid line represents the fitted linear regression line, and the grey shaded area indicates the 95% confidence interval for the mean predicted outcome.

PASE was also positively associated with handgrip strength (ρ = 0.219; *p* < 0.001), indicating a weak positive association:

Handgrip strength = 0.025 × PASE + 14.486, R^2^ ≈ 4.0% (β = 0.025; 95% CI: 0.014 to 0.035) (see Figure 9).

### 3.7. Age-Stratified Results

Younger-old (60–74 years; n = 492)

**Figure 10:**
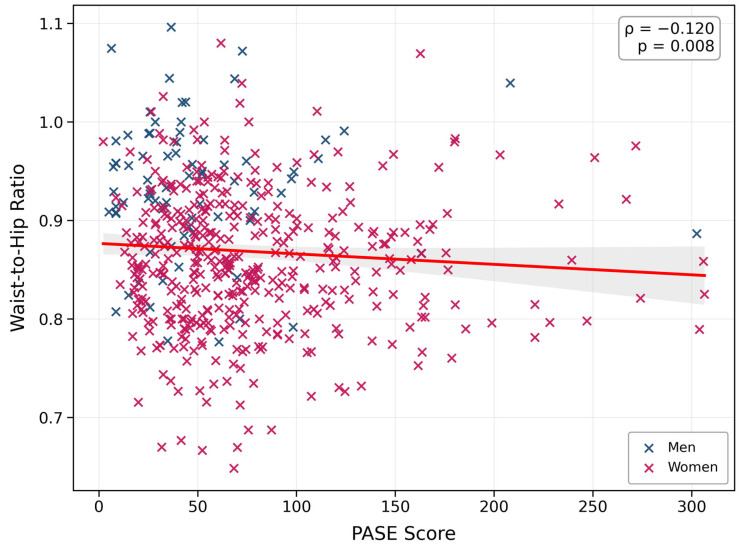
Relationship between physical activity level and waist-to-hip ratio in the elderly (younger-old) group. Note: Each symbol represents an individual observation. The red solid line represents the fitted linear regression line, and the grey shaded area indicates the 95% confidence interval for the mean predicted outcome.

**Figure 11:**
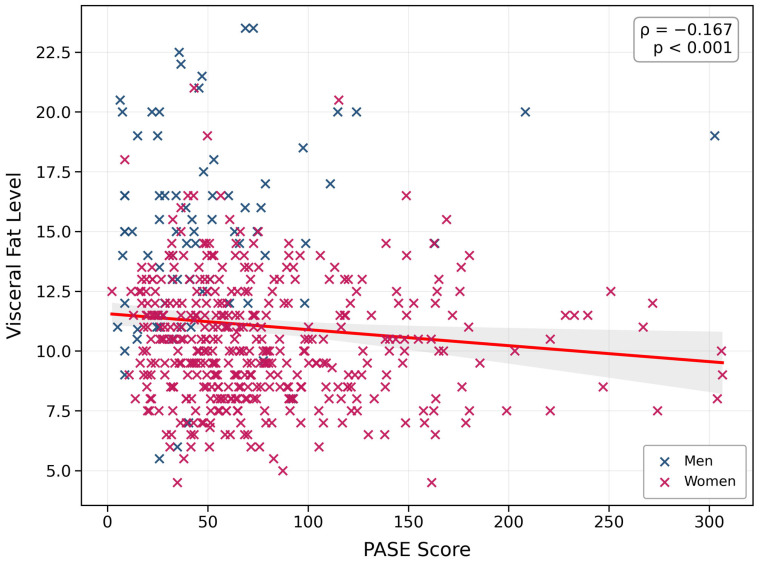
Relationship between physical activity level and visceral fat in the elderly (younger-old) group. Note: Each symbol represents an individual observation. The red solid line represents the fitted linear regression line, and the grey shaded area indicates the 95% confidence interval for the mean predicted outcome.

In this subgroup, PASE correlated inversely with WHR (ρ = −0.120; *p* = 0.008) and visceral fat (ρ = −0.167; *p* < 0.001). Models were:

WHR = −0.00011 × PASE + 0.877, R^2^ ≈ 0.6% (β = −0.00011; 95% CI: −0.00023 to 0.00002) (see Figure 10);

Visceral fat = −0.0067 × PASE + 11.559, R^2^ ≈ 1.2% (β = −0.0067; 95% CI: −0.0121 to −0.0012) (see Figure 11).

Descriptive characteristics for this subgroup are reported in Table 4.

Older-old (≥75 years; n = 116)

**Figure 12:**
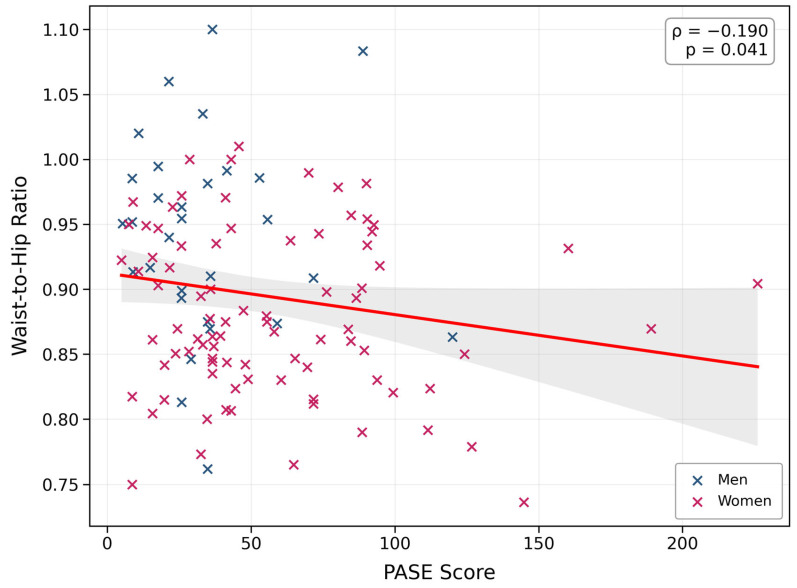
Relationship between physical activity level and waist-to-hip ratio (WHR) in senile-age (older-old) group. Note: Each symbol represents an individual observation. The red solid line represents the fitted linear regression line, and the grey shaded area indicates the 95% confidence interval for the mean predicted outcome.

**Figure 13:**
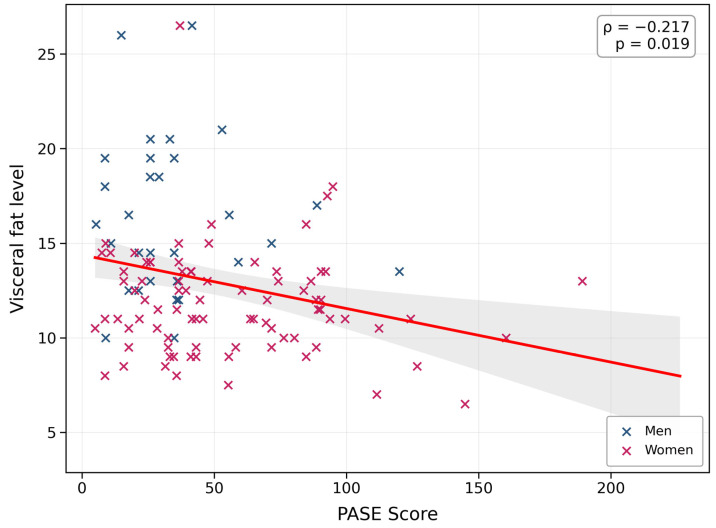
Relationship between physical activity level and visceral fat in senile-age (older-old) olroup. Note: Each symbol represents an individual observation. The red solid line represents the fitted linear regression line, and the grey shaded area indicates the 95% confidence interval for the mean predicted outcome.

In the oldest-old subgroup (≥75 years; *n* = 116), PASE correlated inversely with WHR (ρ = −0.190; *p* = 0.041) and visceral fat (ρ = −0.217; *p* = 0.019). Models were:

WHR = −0.00032 × PASE + 0.912, R^2^ ≈ 2.9% (β = −0.00032; 95% CI: −0.00066 to 0.00002) (see Figure 12);

Visceral fat = −0.028 × PASE + 14.380, R^2^ ≈ 8.2% (β = −0.028; 95% CI: −0.046 to −0.011) (see Figure 13).

**Figure 14:**
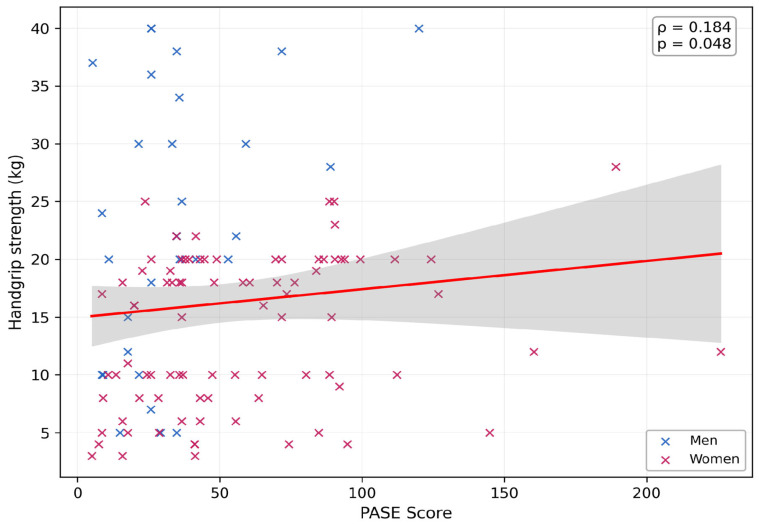
Relationship between physical activity level in senile-age (older-old) individuals and muscle strength values. Note: Each symbol represents an individual observation. The red solid line represents the fitted linear regression line, and the grey shaded area indicates the 95% confidence interval for the mean predicted outcome.

PASE was also positively associated with handgrip strength (ρ = 0.184; *p* = 0.048):

Handgrip strength = 0.022 × PASE + 15.203, R^2^ ≈ 0.9% (β = 0.022; 95% CI: −0.021 to 0.065) (see Figure 14).

Detailed subgroup characteristics are reported in Table 5.

### 3.8. Summary of Key Findings

Overall, most participants reported moderate total physical activity levels—driven primarily by walking and household tasks—while structured moderate-to-vigorous or strength-based exercise occurred infrequently. Across sex and age strata, higher activity levels were consistently associated with lower central adiposity (visceral fat, WHR) and better muscle strength (handgrip), with effect sizes modest as expected for cross-sectional data.

Contextually, independently living attendees of Active Longevity Centers (with greater functional autonomy and mobility) more often engaged in accessible activities (walking, light recreational tasks, household work), whereas Social Service Center residents (nursing home-like support, reduced independent ambulation) participated less in higher-intensity or specialized activities. These patterns underscore the importance of habitual, accessible activities (e.g., walking and household work) as scalable avenues to maintain favorable functional and metabolic profiles in later life.

## 4. Discussion

The present study contributes regional evidence on physical activity patterns and their associations with body composition and functional indicators among urban-dwelling older adults in Central Asia. Higher self-reported physical activity was associated with lower visceral fat, lower waist-to-hip ratio, and greater handgrip strength. Although the magnitude of these associations was generally weak and the regression models had low explanatory capacity, their consistent direction across the total sample and subgroup analyses supports their relevance as hypothesis-generating findings rather than evidence of strong clinical effects.

Across the full sample, higher PASE scores were inversely associated with visceral fat (ρ = −0.214; *p* < 0.001) and waist-to-hip ratio (ρ = −0.154; *p* < 0.001). Sex- and age-stratified analyses showed similar patterns, although some associations differed by subgroup. Among women, higher physical activity was associated with lower BMI, lower total body fat, lower visceral fat, and greater handgrip strength, whereas among men the clearest association was observed with handgrip strength. In the older-old group, physical activity was also positively associated with handgrip strength. These findings suggest that higher physical activity is related to more favorable body composition and functional indicators in older adults. However, the observed associations were weak in magnitude and should be interpreted with caution. Because of the cross-sectional design, reverse causality cannot be excluded. Therefore, these results should be considered descriptive associations, and longitudinal studies are needed to clarify the temporal and causal direction of these relationships.

The practical significance of these findings should be interpreted with caution. Although several associations reached statistical significance, the effect sizes were small and the explanatory capacity of the regression models was low. In the overall sample, PASE explained approximately 2.8% of the variance in visceral fat and approximately 1.2% of the variance in waist-to-hip ratio. Similarly, in subgroup analyses, R^2^ values generally remained low, indicating that physical activity accounted for only a small proportion of variability in body composition and functional indicators. Therefore, these findings should not be interpreted as evidence of strong clinical effects or as sufficient for individual-level prediction.

Another important consideration is residual confounding. Chronic diseases, medication use, baseline functional capacity, nutritional status, socioeconomic factors, and living arrangement may influence both physical activity level and body composition indicators. Since these variables were not included in the present analysis, the observed associations cannot be interpreted as independent effects of physical activity. Instead, they should be viewed as crude descriptive relationships that may partly reflect differences in health status, functional reserve, and social conditions among older adults.

These findings are consistent with broader evidence suggesting that habitual movement is linked to healthier cardiometabolic and functional profiles in older adults.

When interpreting the observed correlations between PASE score and body composition or functional indicators, it is important to consider the specific characteristics of the PASE instrument. Compared with accelerometry, which mainly captures movement patterns such as steps, movement intensity, and sedentary time, PASE provides a broader multidomain assessment of physical activity in older adults. This can be considered a methodological advantage when studying older adults, whose activity is often not limited to structured exercise. However, because PASE is based on self-report over the previous seven days, recall bias, inaccurate reporting, and possible overestimation of activity levels cannot be excluded. Therefore, the observed associations should be interpreted in the context of both the multidomain strength and the self-reported nature of the PASE assessment.

Despite these limitations, the study provides useful regional baseline data and identifies walking and household activities as major contributors to daily activity among older adults in Astana. These findings provide a basis for future longitudinal studies and community-based physical activity programs using objective activity measures.

## 5. Conclusions and Limitations

### 5.1. Conclusions

This cross-sectional study provides regional evidence on physical activity patterns and their associations with anthropometric measures, body composition parameters, and handgrip strength among urban-dwelling older adults in Astana, Kazakhstan. Higher physical activity levels were associated with selected bioelectrical impedance parameters, WHR, and handgrip strength, while walking and household activities were the main contributors to daily physical activity.

Although the study design does not allow causal inference and the observed associations were limited in magnitude, the findings provide useful baseline evidence from an underrepresented region. These data may inform the development of feasible and accessible physical activity strategies and support future longitudinal and intervention studies using objective physical activity measures among older adults in Central Asia.

### 5.2. Strengths

This study has several key strengths. First, it includes a large and diverse sample (*n* = 608) spanning both community-dwelling older adults and residents of long-term care facilities, enabling stratified analyses across functional groups. Second, it uses a validated, widely applied instrument (PASE) to assess physical activity, allowing comparability with international aging research. Third, the study incorporates detailed anthropometric and body composition assessments, including visceral fat, skeletal muscle mass, and handgrip strength—thus capturing multiple dimensions of “healthy aging” beyond simple weight-based metrics. Fourth, this is one of the few studies to characterize physical activity among older adults in Central Asia, a region with limited gerontological data.

### 5.3. Public Health Implications

These findings have meaningful implications for public health planning, geriatric care, and community program development in Central Asia. The observed association between daily movement—primarily walking and household tasks –and improved metabolic and functional profiles suggests that population-level strategies need not rely solely on formal exercise programs. Instead, interventions that promote accessible, low-barrier forms of physical activity may support functional maintenance and healthier activity patterns. This is particularly important in urban settings, where walkability, access to safe communal spaces, and transportation infrastructure influence daily mobility.

Community-based, government funded Active Longevity Centers in Kazakhstan appear to provide a valuable platform for supporting healthy aging; expanding such community-based programs could enhance physical activity opportunities for functionally independent older adults. Conversely, Social Service Centers, which house individuals with lower mobility, may require adapted, supervised, low-intensity movement programs integrated into daily care routines to help mitigate the risk of accelerated functional decline.

At a policy level, the results support integrating physical activity promotion into healthy aging strategies, chronic disease prevention initiatives, and long-term care guidelines. Future programs could evaluate whether small increases in daily movement, supported through structured programs, environmental design, or caregiver training, are associated with meaningful changes in metabolic and functional outcomes.

### 5.4. Limitations

As with any cross-sectional community-based study, several methodological considerations should be taken into account when interpreting the findings. The study design does not allow causal inference, and the observed associations may be bidirectional. Physical activity was assessed partly through self-report, which may introduce recall bias or over- or under-reporting. Body composition estimates derived from bioelectrical impedance analysis (BIA), although practical and non-invasive, may be influenced by hydration status and device-specific algorithms, particularly for visceral fat indices. The sample was recruited from a single urban area, which may limit generalizability to rural populations or other regions of Central Asia. Finally, participation in structured moderate-to-vigorous or resistance exercise was low, restricting the ability to evaluate relationships across the full intensity spectrum.

Several limitations should be acknowledged. First, participants were recruited using convenience sampling rather than random sampling, which may limit the representativeness of the findings. Second, the sample was predominantly female, which may reduce the generalizability of the results to older men. Third, most participants were recruited from Active Longevity Centers, and these individuals may represent a healthier, more mobile, and more socially active subgroup of older adults. Therefore, potential selection bias cannot be excluded. Fourth, the sample included both community-dwelling older adults and nursing home residents; however, the aim of the present study was not to compare participants from different types of institutions. Accordingly, institution type was not included as a covariate in the statistical models. Since these groups may differ in functional status, health condition, and habitual physical activity level, the lack of adjustment for institution type and other potential confounding factors should be considered when interpreting the results. Therefore, the observed associations should be regarded as descriptive relationships rather than independent effects. Finally, no formal cognitive screening tool was used, although participants were required to understand the study procedures, provide informed consent, and complete the questionnaire independently or with assistance from a researcher.

The regression analyses were based on simple crude models and did not adjust for potential confounding factors such as age, sex, BMI, chronic disease burden, socioeconomic status, or institution type. In addition, the models had low explanatory capacity, indicating that PASE score explained only a small proportion of the variability in the analyzed outcomes. Therefore, the observed associations should be interpreted as statistically significant but weak descriptive relationships, rather than as independent or clinically determinative effects.

Despite these limitations, the study provides foundational regional data on the physical activity–health profile of older adults in an underrepresented region and offers useful insights for practice and policy.

## Figures and Tables

**Table 1:** Anthropometric and body composition characteristics of study participants.

Indicator	Category	Value	95% CI/Q1–Q3	*n*
Type of institution	Active Longevity Center	89.8%	87.1–92.1	546
	Social Service Center	10.2%	7.9–12.9	62
Age (years)		68.0	65.0–73.0	608
Systolic blood pressure (mmHg)		140.0	125.0–152.0	608
Diastolic blood pressure (mmHg)		85.0	78.75–93.0	608
WHR (M ± SD)		0.87 ± 0.07	0.87–0.88	608
BMI (kg/m^2^)		27.73 ± 4.80	27.35–28.12	608
BMI categories	Severe underweight	0.00%	0.0–0.6	0
	Underweight	1.48%	0.8–2.8	9
	Normal	28.45%	25.0–32.2	173
	Pre-obesity	39.80%	36.0–43.7	242
	Obesity class I	24.51%	21.3–28.1	149
	Obesity class II	5.26%	3.8–7.3	32
	Obesity class III	0.49%	0.2–1.4	3
Total body fat (%)		37.8	32.6–42.6	608
Total body fat—categories	Deficit	1.6%	0.8–3.0	10
	Normal	33.9%	30.1–37.8	206
	Excess	27.0%	23.5–30.7	164
	Obesity	37.5%	33.6–41.5	228
Visceral fat (units)		11.0	9.0–13.0	608
Visceral fat—categories	Normal	70.4%	66.6–74.0	428
	Excess	29.6%	26.0–33.4	180
Skeletal muscle mass (kg)		40.20	36.80–44.25	608
Bone mass (kg)		2.00	2.00–2.40	608
Total body water (%)		44.70	41.60–48.12	608
Handgrip strength (kg)		19.0	10.0–21.0	608
Basal metabolic rate (kcal/day)		1286.5	1185.0–1402.25	608
Metabolic age (years)		66.0	57.0–77.0	608

**Note:** Obesity classification according to the World Health Organization.

**Table 2:** Anthropometric and body composition characteristics of men.

Indicator	Category	Value	95% CI/Q1–Q3	*n*
Age		71.15 ± 5.72	70.06–72.23	109
WHR (M ± SD)		0.93 ± 0.07	0.92–0.94	109
BMI (kg/m^2^) (M ± SD)		26.37 ± 4.52	25.52–27.22	109
BMI categories	Severe underweight	0.90%	0.2–5.0	1
	Underweight	1.80%	0.5–6.4	2
	Normal	37.60%	29.1–47.0	41
	Pre-obesity	38.50%	29.9–47.9	42
	Obesity class I	16.50%	10.7–24.6	18
	Obesity class II	4.60%	2.0–10.3	5
	Obesity class III		0.0–3.4	0
Total body fat (%) (M ± SD)		28.50 ± 6.94	27.20–29.80	109
Total body fat categories	Deficit	0.90%	0.2–5.0	1
	Normal	32.10%	24.1–41.4	35
	Excess	26.60%	19.2–35.6	29
	Obesity	40.40%	31.6–49.8	44
Visceral fat (units) (M ± SD)		15.26 ± 4.09	14.49–16.03	109
Visceral fat categories	Normal	27.50%	20.0–36.6	30
	Excess	72.50%	63.4–80.0	79
Skeletal muscle mass (kg)		49.3	46.40–54.50	109
Handgrip strength (kg)		30	20.0–37.0	109
Metabolic age (years)		68	58.0–81.0	109
PASE		34.83	24.60–59.05	109
PASE categories	Low	77.98%	69.3–84.7	85
	Moderate	20.18%	13.7–28.7	22
	High	1.83%	0.5–6.4	2

**Note:** Obesity classification according to the World Health Organization.

**Table 3:** Anthropometric and body composition characteristics of women.

Indicator	Category	Value	95% CI/Q1–Q3	*n*
Age		68	64.0–73.0	499
WHR (M ± SD)		0.88 ± 0.07	0.87–0.89	499
BMI (kg/m^2^) (M ± SD)		27.97 ± 4.59	27.57–28.38	499
BMI categories	Severe underweight		0.0–0.8	0
	Underweight	1.20%	0.6–2.6	6
	Normal	26.45%	22.8–30.5	132
	Pre-obesity	39.88%	35.7–44.2	199
	Obesity class I	26.25%	22.6–30.3	131
	Obesity class II	5.81%	4.1–8.2	29
	Obesity class III	0.40%	0.1–1.4	2
Total body fat (%) (M ± SD)		38.5 ± 6.0	38.0–39.1	499
Total body fat categories	Deficit	1.40%	0.6–2.9	7
	Normal	32.30%	28.4–36.5	161
	Excess	25.70%	22.0–29.8	128
	Obesity	40.60%	36.2–45.1	203
Visceral fat (units) (M ± SD)		10.4 ± 2.6	10.2–10.7	499
Visceral fat categories	Normal	72.10%	68.0–75.9	360
	Excess	27.90%	24.1–32.0	139
Skeletal muscle mass (kg)		38.8	36.2–41.9	499
Handgrip strength (kg)		17	10.0–20.0	499
Metabolic age (years)		65	55.0–76.0	499
PASE		61.45	37.92–92.65	499
PASE categories	Low	20.64%	17.3–24.4	103
	Moderate	70.94%	66.8–74.8	354
	High	8.42%	6.3–11.2	42

**Note:** Obesity classification according to the World Health Organization.

**Table 4:** Anthropometric and body composition characteristics of younger-old respondents.

Indicator	Category	Value	95% CI/Q1–Q3	*n*
Age range (years)		60–74	N/A	492
WHR (M ± SD)		0.87 ± 0.07	0.86–0.88	492
BMI (kg/m^2^) (M ± SD)		27.71 ± 4.93	27.28–28.15	492
BMI categories	Severe underweight	0%	0.0–0.8	0
	Underweight	1.83%	1.0–3.4	9
	Normal	28.86%	25.0–33.0	142
	Pre-obesity	39.23%	35.0–43.6	193
	Obesity class I	24.39%	20.8–28.4	120
	Obesity class II	5.08%	3.5–7.4	25
	Obesity class III	0.61%	0.2–1.8	3
Total body fat (%) (M ± SD)		37.0 ± 7.42	36.34–37.65	492
Total body fat categories	Deficit	1.60%	0.5–2.7	8
	Normal	34.80%	30.5–39.0	171
	Excess	27.00%	23.1–31.0	133
	Obesity	36.60%	32.3–40.8	180
Visceral fat (units)		10.5	8.5–13.0	492
Visceral fat categories	Normal	74.40%	70.5–78.2	366
	Excess	25.60%	21.8–29.5	126
Metabolic age (years)		64	55.0–77.0	492
Handgrip strength (kg)		20	12.0–22.0	492
PASE		60.3	36.6–92.6	492
PASE categories	Low	31.71%	27.8–35.9	156
	Moderate	60.57%	56.2–64.8	298
	High	7.72%	5.7–10.4	38

**Note:** Obesity classification according to the World Health Organization.

**Table 5:** Anthropometric and body composition characteristics of senile-age (older-old) respondents.

Parameter	Category	Value	95% CI/Q1–Q3	*n*
Age range (years)		≥75	N/A	116
WHR (M ± SD)		0.90 ± 0.07	0.88–0.91	116
BMI (kg/m^2^) (M ± SD)		27.82 ± 4.33	27.02–28.61	116
BMI categories	Severe underweight	0%	0.0–3.2	0
	Underweight	0.00%	0.0–3.2	0
	Normal	26.72%	19.5–35.4	31
	Pre-obesity	42.24%	33.6–51.3	49
	Obesity class I	24.14%	17.3–32.7	28
	Obesity class II	6.90%	3.5–13.0	8
	Obesity class III	0.00%	0.0–3.2	0
Total body fat (%) (M ± SD)		36.67 ± 7.24	35.34–38.00	116
Total body fat categories	Deficit	1.72%	0.5–6.1	2
	Normal	30.17%	22.6–39.1	35
	Excess	26.72%	19.5–35.4	31
	Obesity	41.38%	32.8–50.5	48
Visceral fat (units) (M ± SD)		12.92 ± 3.84	12.21–13.62	116
Visceral fat categories	Normal	53.45%	44.4–62.3	62
	Excess	46.55%	37.7–55.6	54
Metabolic age (years)		69	62.0–80.0	116
Skeletal muscle mass (kg) (M ± SD)		41.08 ± 6.39	39.90–42.25	116
ALMI (M ± SD)		16.57 ± 1.84	16.23–16.91	116
Handgrip strength (kg)		17.5	10.0–20.0	116
PASE		38.6	25.8–72.1	116
PASE categories	Low	27.59%	20.3–36.3	32
	Moderate	67.24%	58.3–75.1	78
	High	5.17%	2.4–10.8	6

**Note:** Obesity classification according to the World Health Organization.

## Data Availability

The data are not publicly available due to privacy restrictions.

## Abbreviations

The following abbreviations are used in this manuscript:

BMI	Body mass index
VF	Visceral fat
WC/HC	Waist and hip circumference
WHR	Waist-to-hip ratio
